# Administration of a Histone Deacetylase Inhibitor into the Basolateral Amygdala Enhances Memory Consolidation, Delays Extinction, and Increases Hippocampal BDNF Levels

**DOI:** 10.3389/fphar.2017.00415

**Published:** 2017-06-28

**Authors:** Fernanda E. Valiati, Mailton Vasconcelos, Martina Lichtenfels, Fernanda S. Petry, Rosa M. M. de Almeida, Gilberto Schwartsmann, Nadja Schröder, Caroline B. de Farias, Rafael Roesler

**Affiliations:** ^1^Department of Pharmacology, Institute for Basic Health Sciences, Federal University of Rio Grande do SulPorto Alegre, Brazil; ^2^Cancer and Neurobiology Laboratory, Experimental Research Center, Clinical Hospital, Federal University of Rio Grande do SulPorto Alegre, Brazil; ^3^Institute of Psychology, Federal University of Rio Grande do SulPorto Alegre, Brazil; ^4^Department of Internal Medicine, Faculty of Medicine, Federal University of Rio Grande do SulPorto Alegre, Brazil; ^5^Neurobiology and Developmental Biology Laboratory, Faculty of Biosciences, Pontifical Catholic University of Rio Grande do SulPorto Alegre, Brazil; ^6^Children’s Cancer InstitutePorto Alegre, Brazil

**Keywords:** histone deacetylase, brain-derived neurotrophic factor, amygdala, hippocampus, memory extinction, memory consolidation

## Abstract

Gene expression related to the formation and modification of memories is regulated epigenetically by chromatin remodeling through histone acetylation. Memory formation and extinction can be enhanced by treatment with inhibitors of histone deacetylases (HDACs). The basolateral amygdala (BLA) is a brain area critically involved in regulating memory for inhibitory avoidance (IA). However, previous studies have not examined the effects of HDAC inhibition in the amygdala on memory for IA. Here we show that infusion of an HDAC inhibitor (HDACi), trichostatin A (TSA), into the BLA, enhanced consolidation of IA memory in rats when given at 1.5, 3, or 6 h posttraining, but not when the drug was infused immediately after training. In addition, intra-BLA administration of TSA immediately after retrieval delayed extinction learning. Moreover, we show that intra-BLA TSA in rats given IA training increased the levels of brain-derived neurotrophic factor in the dorsal hippocampus, but not in the BLA itself. These findings reveal novel aspects of the regulation of fear memory by epigenetic mechanisms in the amygdala.

## Introduction

In inhibitory avoidance (IA), a type of fear-motivated conditioning, a new memory is formed after a single training trial, and the behavioral outcome of previously formed memories can be modified upon recall through extinction or reconsolidation. These processes are mediated and regulated by a range of neurotransmitter and neuropeptide receptors, intracellular protein kinase signaling pathways, and transcription factors, resulting in changes in gene transcription. Brain areas critically involved in mediating or regulating the formation and extinction of IA memory include the dorsal hippocampus and the basolateral amygdala (BLA; McGaugh, 2000; Roesler and McGaugh, 2010; Roesler and Schröder, 2011; Roozendaal and McGaugh, 2011; Alberini and Kandel, 2014; Furini et al., 2014; Roesler et al., 2014a; Izquierdo et al., 2016). The BLA is proposed to interact with the hippocampus and related structures to enhance the consolidation of memory for events that trigger fear or aversiveness (McGaugh, 2002; McIntyre et al., 2003).

Gene expression related to memory consolidation is regulated epigenetically by chromatin remodeling, post-translational DNA modifications, and small RNAs (Levenson and Sweatt, 2005; Barrett and Wood, 2008; Mikaelsson and Miller, 2011; Gräff and Tsai, 2013; Landry et al., 2013). Modifications in chromatin state influence the access of the transcriptional machinery to the genome. It is now well established that DNA methylation and histone acetylation are crucial epigenetic processes influencing long-term fear memory (Barrett and Wood, 2008; Mikaelsson and Miller, 2011; Gräff and Tsai, 2013). Histone deacetylase (HDAC) proteins deacetylate N-terminal lysine residues in histones, leading to a more compact chromatin structure and reduced gene transcription (Kouzarides, 2007).

HDAC inhibitors (HDACis) are the most widely investigated pharmacological agents modulating epigenetic processes. Administration of HDACis lead to increased acetylation and enhanced gene expression in neurons, resulting in a facilitation of synaptic plasticity as well as formation and extinction of fear conditioning (Levenson et al., 2004; Lattal et al., 2007; Vecsey et al., 2007; Bredy and Barad, 2008; Stafford et al., 2012). Acute systemic or intrahippocampal administration of HDACis enhances IA memory consolidation and rescues IA deficits related to aging or models of memory impairment (Silva et al., 2012; Blank et al., 2014, 2015, 2016; Sharma et al., 2015; Petry et al., 2016). Epigenetic alterations in the lateral amygdala, including increased histone H3 acetylation, are involved in the formation and reconsolidation of memory for auditory fear conditioning in rats. Intraamygdala infusion of the HDACi trichostatin A (TSA) enhances both the consolidation and reconsolidation of auditory fear memory (Maddox and Schafe, 2011; Monsey et al., 2011). Increased H3 acetylation in the amygdala is also related to accelerated extinction of auditory fear conditioning in mice after a systemic injection of an HDACi (Itzhak et al., 2012). However, previous studies have not examined the effects of HDAC inhibition in the amygdala on memory for IA. In the present study, we investigated the effects of TSA infused into the BLA at several time points after training, or immediately after retrieval, on the consolidation and extinction of IA memory in rats. Given the reported interactions between the BLA and dorsal hippocampus mentioned above, the enhancing effect of HDAC inhibitors on the expression of brain-derived neurotrophic factor (BDNF; Wu et al., 2008), and the role of hippocampal BDNF in promoting memory for IA (Chen et al., 2012), we also verified whether intra-BLA infusion of TSA resulted in an increase in hippocampal BDNF levels.

## Materials and Methods

### Animals

Adult male Wistar rats (220–350 g at time of surgery) were obtained from the institutional breeding facility (CREAL, ICBS, UFRGS) and maintained at the university hospital animal research facility (UEA, CPE-HCPA). Animals were housed four per cage in plastic cages with sawdust bedding and maintained on a 12 h light/dark cycle at a room temperature of 22 ± 2°C. The rats were allowed *ad libitum* access to standardized pellet food and water. All experiments took place during the light phase, between 8 AM and 5 PM.

### Surgery

Rats were implanted under anesthesia with isoflurane (vaporized in 100% oxygen, at a dose of 5% for induction and 2% for maintenance, in a fraction of 0.5 l/min) with bilateral 14-mm, 23-gauge guide cannulae aimed 1.0 mm above the BLA, as described previously (Roesler et al., 2004; Jobim et al., 2012). Coordinates (anteroposterior, -2.8 mm from bregma; mediolateral, ±4.8 mm from bregma; ventral, -7.5 mm from skull surface) were obtained from the atlas of Paxinos and Watson (2007). Rats were allowed to recover at least 5 days after surgery before behavioral training.

### Inhibitory Avoidance

Single-trial step-down IA was used as an established model of fear-motivated conditioning memory, where the animals learn to associate a location in the training apparatus (a grid floor) with an aversive stimulus (footshock). The general procedures for IA behavioral training and retention tests were described in previous reports (Jobim et al., 2012; Blank et al., 2014). The IA training apparatus was a 50 cm × 25 cm × 25 cm acrylic box (Albarsch, Porto Alegre, Brazil) with a floor composed of parallel caliber stainless steel bars (1 mm diameter) spaced 1 cm apart. A 7-cm wide, 2.5-cm high platform was placed on the floor of the box against one wall.

On training trials, rats were placed on the platform and their latency to step down on the grid with all four paws was measured with a digital chronometer. Immediately after stepping down on the grid, rats received a 0.4-mA, 3.0-s footshock and then removed from the apparatus immediately afterward. The first retention test trial was given 24 h after training by placing the rats on the platform and recording their latencies to step down. No footshock was presented during retention test trials. Step-down latencies on the retention test trial (maximum 300 s) were used as a measure of IA memory retention.

For IA extinction, rats were returned daily to the IA training context without footshock for 6 days as described previously (Roesler et al., 2014b; Petry et al., 2016). Rats that did not step down to the grid floor within 300 s during the first 24 h retention/extinction test trial were gently led by experimenter to the grid floor. Rats were given a 0.3 mA reminder footshock at the end of the fifth test, followed by an additional retention test 24 h later (Tronel and Alberini, 2007; Roesler et al., 2014b).

### Drug Infusions

The general procedures for BLA infusions were described in previous reports (Jobim et al., 2012; Pedroso et al., 2013). At the time of infusion, a 27-gauge infusion needle was fitted into the guide cannula. The tip of the infusion needle protruded 1.0 mm beyond the guide cannula and was aimed at the BLA. Drug or vehicle was infused during a 30-s period. The infusion needle was left in place for an additional minute to allow diffusion of the drug away from the needle tip.

In the experiment to examine the memory consolidation, rats received a bilateral 0.5-μl infusion of TSA (Sigma-Aldrich, St. Louis, United States; 22 mM) dissolved in 50% ethanol in saline (vehicle, VEH; Vecsey et al., 2007) into the BLA at different times after IA training. Different groups of rats were used for each infusion time point. Control animals received VEH in the same condition. In the memory extinction experiment, rats received a bilateral 0.5-μl infusion of TSA (22 mM) or VEH immediately after the first test trial. The TSA dose was chosen on the basis of previous findings from our group showing that it enhanced IA memory consolidation when given into the dorsal hippocampus (Blank et al., 2014). Drug solutions were prepared freshly before each experiment.

### Measurement of BDNF Levels

A separate group of rats was given one IA training trial as described above, followed immediately by an intra-BLA infusion of TSA (22 mM) or VEH. Four hours later, the rats were sacrificed by decapitation, their brains were removed and the BLA and hippocampus were quickly dissected out, immediately snap-frozen in liquid nitrogen and stored at -80°C until BDNF measurement. The posttraining time for BDNF measurement was chosen on the basis of a previous study showing that hippocampal BDNF levels increased 4 h after learning (Goulart et al., 2010). BLA and hippocampal BDNF was measured as described previously (Kauer-Sant’Anna et al., 2007; Goulart et al., 2010), using sandwich enzyme-linked immunosorbent assay (ELISA) commercial kits according to the manufacturer’s instructions (ChemiKine^TM^, CYT306, Merck Millipore, Temecula, United States). Briefly, samples were homogenized in phosphate-buffered solution with 1 mM phenylmethylsulfonyl fluoride and 1 mM ethyleneglycoltetraacetic acid. Microtiter plates (96-well flat-bottom) were coated for 24 h with the samples diluted 1:2 in sample diluents and the standard curve ranged from 7.8 to 500 pg/ml of BDNF. The plates were then washed four times with wash buffer and a monoclonal anti-BDNF rabbit antibody (1:1000) was added to each well and incubated for 3 h at room temperature. After washing, a peroxidase-conjugated anti-rabbit antibody (horseradish peroxidase enzyme; 1:1000) was added to each well and incubated for 1 h at room temperature. After addition of streptavidin enzyme, substrate (3,3′,5,5′-tetramethylbenzidine) and stop solution, the amount of BDNF was determined by absorbance at 450 nm in a spectrophotometer. Total protein was measured using the Bradford’s method with bovine serum albumin as the standard.

### Histology

A 0.5-μl infusion of a 4% methylene blue solution was infused into the cannulae 24–48 h after the end of behavioral testing. Rats were killed by decapitation 15 min later, and their brains were removed and stored in 10% formalin for at least 72 h. At least 24 h before sectioning, brains were placed in a 20% sucrose solution in water for cryoprotection. Coronal sections of 50 μm were cut on a cryostat, mounted on gelatin-coated slides, stained with hematoxylin and eosin and examined under light microscopy. The extension of the methylene blue dye was taken as indicative of diffusion of the drugs previously given each rat (Roesler et al., 2004; Jobim et al., 2012). Rats with incorrect cannula placements were excluded from the statistical analyses.

### Statistics

Non-parametric tests were used to analyze retention test latencies because of the 300-s cut-off imposed on retention test trials. Training and retention test step-down latencies were analyzed using a Kruskal–Wallis test followed by two-tailed Mann–Whitney *U*-tests. Student’s *t*-tests for independent samples were used for comparisons of BDNF levels between controls and TSA-treated groups within each brain area (BLA or hippocampus). In all comparisons, *p* < 0.05 was considered to indicate statistical significance. All data are shown as mean ± standard error of mean (SEM).

## Results

### Time Course of Consolidation Enhancement by Intra-BLA Administration of TSA

The first set of experiments examined the effects of intra-BLA administration of TSA at different posttraining intervals on IA memory consolidation. Rats were given IA training followed by a bilateral infusion of VEH or TSA (22 mM) into the BLA immediately (VEH, *N* = 10; TSA, *N* = 9), 1.5 h (VEH, *N* = 13; TSA, *N* = 13), 3 h (VEH, *N* = 14; TSA, *N* = 14), or 6 h (VEH, *N* = 13; TSA, *N* = 13) after training. All rats were tested for retention 24 h later after training.

**FIGURE 1:**
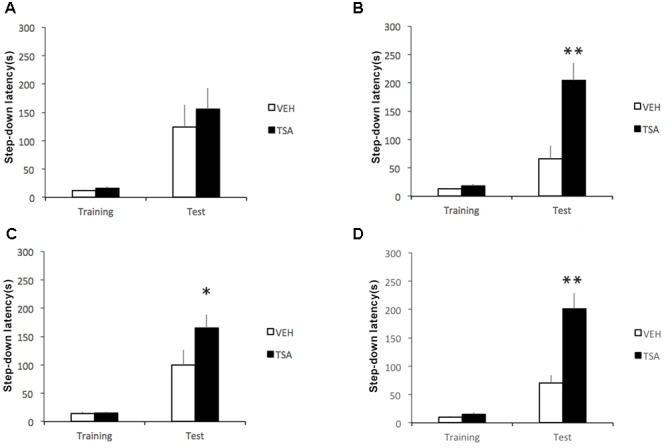
Enhancement of later phases of IA memory consolidation by HDAC inhibition in the amygdala shortly after learning. Rats were given a single IA training trial followed by an infusion of VEH or TSA (22 mM) into the BLA **(A)** immediately, **(B)** 1.5 h, **(C)** 3 h, or **(D)** 6 h after training. Retention was tested 1 day after training in all groups. Data are mean ± SEM latencies to step down; *N* = 9–14 rats per group; ^∗^*p* < 0.05, ^∗∗^*p* < 0.01 compared to respective controls.

Results are shown in **Figure 1**. Infusions given immediately after training had no effect (*p* = 0.43, **Figure 1A**). However, retention test latencies were significantly higher compared to VEH-treated controls in rats infused with TSA at 1.5 (*p* < 0.01, **Figure 1B**), 3 (*p* < 0.05, **Figure 1C**), and 6 (*p* < 0.01, **Figure 1D**) h posttraining. There were no significant differences between groups in training trial latencies (immediate posttraining infusions, *p* = 0.10; 1.5 h posttraining infusions, *p* = 0.25; 3 h posttraining infusions, *p* = 0.96; 6 h posttraining infusions, *p* = 0.09).

### Intra-BLA Administration of TSA Delays IA Memory Extinction

We then went on to verify whether TSA into the amygdala would affect IA extinction. Rats were given bilateral intra-BLA infusions of VEH (*N* = 11) or TSA (*N* = 12) immediately after the first test trial (Test 1), which served as an extinction training trial. All rats were tested for extinction 1 (Test 2), 2 (Test 3), 3 (Test 4), and 4 (Test 5) days after Test 1. Immediately after Test 5, rats were given a reminder footshock and retention was tested again 1 day later in the absence of footshock.

**FIGURE 2:**
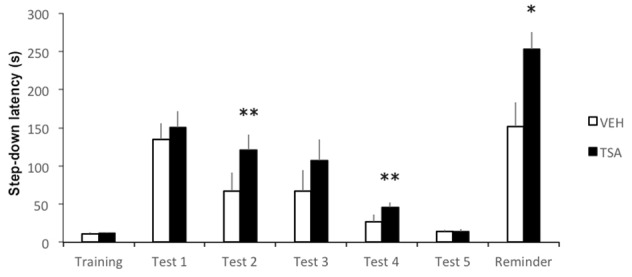
Amygdalar HDAC inhibition delays IA memory extinction. Rats were given a single IA training trial and tested for retention 24 h later (Test 1). This first test trial also served as extinction training. An infusion of VEH or TSA (22 mM) was given into the BLA immediately after Test 1. Retention was tested once daily from days 1 to 5 after Test 1 (Tests 2–5). A mild reminder footshock was given after Test 5 and rats were tested again 1 day later (Reminder). Data are mean ± SEM latencies to step down; VEH, *N* = 11, TSA, *N* = 12; ^∗^*p* < 0.05; ^∗∗^*p* < 0.01 compared to controls.

Results are shown in **Figure 2**. Latencies were significantly higher in TSA-treated rats compared to controls in Test 2 (*p* < 0.01) and Test 4 (*p* < 0.01). The difference in Test 3 latencies did not reach significance (*p* = 0.10), in spite of the apparently higher latency in TSA-treated rats. Both groups reached similar levels of extinction by Test 5 (*p* = 0.68). Overall, the results indicate that TSA delayed extinction. Rats treated with TSA also showed significantly higher latencies than VEH controls after being presented with a reminder footshock (*p* < 0.05), supporting the possibility that the fear response in TSA-treated rats was more resistant to extinction. There were no significant differences between groups in latencies during training (*p* = 0.68).

### TSA Infusion into the BLA Increases BDNF Levels in the Hippocampus But Not Amygdala in IA-Trained Rats

**FIGURE 3:**
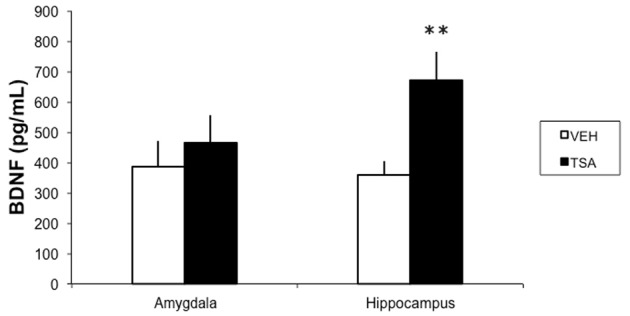
HDAC inhibition in the BLA results in increased BDNF levels in the dorsal hippocampus in rats given IA training. Rats were given a bilateral intra-BLA infusion of VEH or TSA (22 mM) immediately after IA training. Four hours later, they were sacrificed and the BLA and dorsal hippocampus levels were removed for BDNF measured with an ELISA. Data are mean ± SEM pg of BDNF/ml of protein; VEH, *N* = 12, TSA, *N* = 10; ^∗∗^*p* < 0.01 compared to respective controls.

In a separate group of rats given IA training followed by an intra-BLA infusion of VEH (*N* = 12) or TSA (*N* = 10) immediately afterward, and sacrificed for BDNF measurements 4 h later, intra-BLA TSA induced a significant increase in BDNF levels in the hippocampus (*p* < 0.01), but not in the BLA (*p* = 0.85; **Figure 3**).

### Histology

**FIGURE 4:**
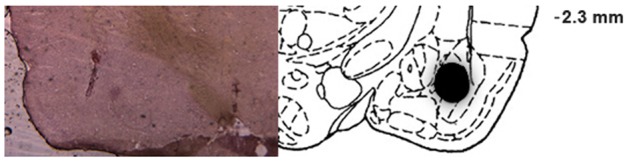
Infusion placements into the BLA. Representative photomicrograph illustrating placement of a cannula and needle tip, and schematic diagram of a coronal section of the rat brain (anteroposterior, –2.8 mm from bregma), adapted from the atlas of Paxinos and Watson (2007), depicting the diffusion of methylene blue in the BLA for rats included in the statistical analysis.

All animals (144 rats) included in the final analysis of IA had cannula bilaterally placed in the BLA. **Figure 4** shows a representative photomicrograph illustrating placement of a cannula and needle tip, as well as a schematic drawing of the diffusion of methylene blue, which indicates infusion placements and spread of drug infusions within the BLA.

## Discussion

Previous studies have shown that administration of HDACis into the amygdala around the time of training or retrieval resulted in enhanced consolidation and reconsolidation of memory for fear conditioning (Maddox and Schafe, 2011; Monsey et al., 2011), and systemic injections of HDACi could also accelerate fear extinction (Lattal et al., 2007; Bredy and Barad, 2008; Itzhak et al., 2012; Stafford et al., 2012). The present study reveals several novel aspects related to the amygdalar HDAC involvement in fear memory. First, we provide the first report of the effects of HDAC inhibition in the amygdala on memory of IA. Second, we show that HDACis can be more effective in enhancing memory when given at later time points during consolidation (up to at least 6 h after training) than when administered before or shortly after learning. Third, we show that HDAC inhibition can impair rather than facilitate fear extinction. Finally, we provide the first evidence that inhibiting HDAC within the amygdala can result in an increase in BDNF levels in the dorsal hippocampus.

In contrast to previous reports (Lattal et al., 2007; Bredy and Barad, 2008; Itzhak et al., 2012; Stafford et al., 2012), we found that treatment with an HDACi delayed rather than facilitated fear extinction. In IA, memory reactivation at the time of testing can initiate either one of two competing processes: further memory strengthening, likely mediated by reconsolidation; or memory extinction (Vianna et al., 2001; Jobim et al., 2012; Pedroso et al., 2013; Furini et al., 2014; Roesler et al., 2014b). It is possible that intra-BLA TSA given after retrieval acts by enhancing reconsolidation to make the original memory more resistant to extinction. The possibility that the original memory for training was stronger in TSA-treated rats is supported by the finding that, compared to controls, they showed an increased avoidance response after exposure to a reminder shock.

Perhaps the most intriguing finding of the present report was that intra-BLA TSA administration led to an increase in BDNF protein content in the dorsal hippocampus, but not in the amygdala. It is well established that BDNF, which acts by activating its receptor, TrkB, resulting in the stimulation of a range of intracellular kinase signaling pathways including phospholipase C/protein kinase C, extracellular signal-regulated protein kinase (ERK)/mitogen-activated protein kinase (MAPK), and phosphatidylinositol 3-kinase, plays a major role in synaptic plasticity and memory formation (Huang and Reichardt, 2003; Minichiello, 2009; Yoshii and Constantine-Paton, 2010). Consolidation of IA memory requires BDNF/TrkB signaling that accompanies protein synthesis in the dorsal hippocampus (Bambah-Mukku et al., 2014), and intrahippocampal administration of an anti-BDNF antibody before training impairs IA retention (Chen et al., 2012). Gene transcription for BDNF is stimulated by HDACis (Wu et al., 2008; Koppel and Timmusk, 2013), and systemic HDACi treatment increases protein levels of BDNF in the rat brain (Kim et al., 2009). In addition, administration of an HDACi into the hippocampus rescues the impairment of IA memory consolidation produced by TrkB inhibition (Blank et al., 2016).

Thus, promoting BDNF expression in the hippocampus could be a crucial mechanism enabling amygdalar HDAC inhibition to enhance different phases of IA memory consolidation. However, TSA infused into the BLA immediately after training, which resulted in an increase in hippocampal BDNF, did not affect retention. It is possible that the increase in BDNF caused by posttraining TSA was related to resistance to extinction, although that BDNF has been shown to induce fear extinction under some circumstances (Peters et al., 2010). Also, what could be the mechanism mediating the increase in hippocampal BDNF after inhibition of amygdalar HDAC? Previous studies have indicated that BLA activity influences gene expression related to IA memory formation in the hippocampus and related brain areas. For instance, BLA activity is required to enable the effects of memory-enhancing agents, including HDACis, given into the hippocampus or entorhinal cortex (Roozendaal and McGaugh, 1997; Roozendaal et al., 1999; Roesler et al., 2002; Blank et al., 2014). Importantly, intra-BLA infusion of a memory-enhancing drug, the beta-adrenoreceptor agonist clenbuterol, resulted in an increase in dorsal hippocampal levels of activity-regulated cytoskeletal protein (Arc, also called Arg 3.1), an immediate-early gene involved in synaptic plasticity and memory consolidation, whereas BLA inactivation by a lidocaine infusion decreased Arc content in the hippocampus (McIntyre et al., 2005; McReynolds et al., 2014). Noradrenaline can enhance histone acetylation (Maity et al., 2016), and possible mechanisms mediating BDNF influences on synaptic plasticity include an upregulation of Arc levels (Yin et al., 2002; Ying et al., 2002). Therefore, the possibility that increased histone acetylation in the BLA can enhance hippocampal BDNF expression is consistent with previously described neurotransmitter and gene expression pathways involved in BLA-hippocampal interactions during memory formation.

Formation of IA memory has been previously shown to involve ERK/MAPK signaling in the hippocampus and BLA 3 h posttraining, but not shortly after training (Walz et al., 2000). Increases in histone H3 acetylation in the amygdala induced by fear conditioning are downstream of ERK/MAPK signaling (Monsey et al., 2011), and BDNF mediates enhancing effects on memory through MAPK activation (Revest et al., 2014). Therefore, stimulation of hippocampal MAPK activity through up-regulation of BDNF induced by intra-BLA TSA arises as another candidate mechanism for the effects observed in our study. It is likely that the enhancing effect of intra-BLA TSA also involves the combined action of several other mechanisms. TSA is a hydroxamic acid containing a functional group that interacts with the critical zinc atom at the base of the catalytic pocket of HDACs, thus inhibiting their activity. In addition to inhibiting class I HDACs, which are localized predominantly to the cell nucleus, TSA also inhibits HDAC6, the main cellular cytoplasmic deacetylase (Bhalla, 2005; Bolden et al., 2006). Moreover, HDACis might display extra-epigenetic effects, such as direct interactions with cytoplasmic cell signaling pathways and acetylation of non-histone proteins (Chen et al., 2005; Glozak et al., 2005).

In summary, the present findings reveal novel aspects of the involvement of amygdalar epigenetic mechanisms in fear memory, by showing that HDAC inhibition in the amygdala can enhance a later phase of consolidation, delay extinction, and possibly act by increasing BDNF levels in the dorsal hippocampus. Our findings raise the exciting possibility that epigenetic manipulations within the BLA affect memory processes by influencing the expression of molecules mediating synaptic plasticity in the hippocampus rather than the amygdala itself.

## Ethics Statement

All experimental procedures were performed in accordance with the Brazilian Guidelines for the Care and Use of Animals in Research and Teaching (DBCA, published by CONCEA, MCTI) and approved by the institutional Animal Care Committee (CEUA-HCPA) under protocol number 140429.

## Author Contributions

FV, GS, NS, CdF, and RR designed the research; FV, MV, ML, FP, and CdF performed experiments; FV analyzed the data; FV, RdA, GS, NS, and RR interpreted and discussed the data; GS, RdA, NS, and RR provided materials; FV and RR wrote the paper; FV, MV, ML, FP, RdA, GS, NS, CdF, and RR revised the manuscript.

## Conflict of Interest Statement

The authors declare that the research was conducted in the absence of any commercial or financial relationships that could be construed as a potential conflict of interest.
